# Health behaviors and personality in burnout: a third dimension

**DOI:** 10.3402/meo.v20.28187

**Published:** 2015-09-11

**Authors:** Osama M. Mustafa

**Affiliations:** College of Medicine, Alfaisal University, Riyadh, Saudi Arabia

**Keywords:** health behavior, personality, intelligence, predictors, burnout, depression, stress, professional, students, medical

## Abstract

The high prevalence of burnout among healthcare professionals warrants a thorough examination aimed at improving the current understanding of its predictors and preventive measures. Cecil et al. have underscored the alarming prevalence of burnout among medical students and assessed its association with demographic, lifestyle, and behavioral factors. Of interest, health behaviors were found to be predictive of burnout. The study suggests certain behaviors (such as high physical activity) to be protective, and thus, calls for their establishment early in college life to prevent the development of this professionally-disabling mental state. Although the adoption of advisable health behaviors may independently reduce the risk of burnout, recognition of the existence and influence of closely related factors allows for an enhanced understanding and a greater precision for any conclusions to be made. Personality, through deductive and inductive reasoning, is likely to exert significant influence on both the student's behavior and his/her susceptibility to burnout. Thus, with personality representing – in and of itself – a principal model for prediction of burnout risk, controlling for personality traits when addressing health behaviors’ influence *per se* on burnout is essential.

In a former article, Cecil and colleagues addressed a phenomenon with pronounced presence across all healthcare educational and training strata, highlighting its particular relevance to medical students (1). Specifically, their study provides an informative perspective on burnout and its potential association with health behaviors in a sample of undergraduate medical students. While attempting to identify potential predictors, physical activity was found to be the most predictive of burnout component scores across all investigated lifestyle and health-behavior variables, with increased physical activity being significantly associated with high personal achievement (PA) and low emotional exhaustion (EE) scores. Of note, it was concluded that making healthier lifestyle choices should be encouraged in early college life to prevent the development of burnout. One important question, however, should be raised here: Is unhealthy lifestyle a true precipitator of burnout or a mere reflector of one's susceptibility towards this persistent negative mental state?

## Plausibility and methodological limitations

With an observational, cross-sectional design, there is a limited capacity to establish a cause-and-effect relationship. Certainly, existing evidence indicates the potential contribution of physical activity to the improvement of mental health (2–5). Therefore, the ability of physical activity to augment burnout reduction efforts may be deemed plausible. Additionally, health behaviors’ predictive capacity of burnout – as suggested by Cecil et al.'s work – may imply a causative relationship and may accordingly propose modifications of health behaviors as possible interventions to prevent burnout. Although this could be true, it should be noted that conclusions drawn from the cited study are bound to restrictions resulting from the inability to establish temporal precedence, lack of study-sample control, and absence of objective measurements of the addressed predictors (e.g., self-reported vs. measured physical activity).

## The opposing premise

In its essence, burnout results from the accumulation of emotional disturbances, perception of low self-capacity, and maladaptation – all of which are elicited by stressors and subsequently culminate in suboptimal functioning (6). This can initiate a cycle of continuous emotional disturbance that fosters further deterioration of functionality and performance. Such a suboptimal mental state may affect rational decision-making and drive behavior toward unhealthy acts such as smoking, drinking, and inactivity (7, 8). In fact, burnout has been implicated in extremes of health-destructive behaviors, such as drug abuse and suicide, even when depressive symptoms are controlled for (9, 10). Repercussions of the psychological distress of burnout may well extend beyond health behaviors to erode the student's professional behavior, leading to dishonesty, lack of empathy, and deranged ethical attitudes (11, 12). Accordingly, it can be argued that disturbed health behaviors may be a result, rather than a cause, of burnout. Given the possibility of the existence of undesirable health behaviors as a cause and/or a consequence of burnout, are we facing a paradox?

## The missing perspective

**Fig. 1:**
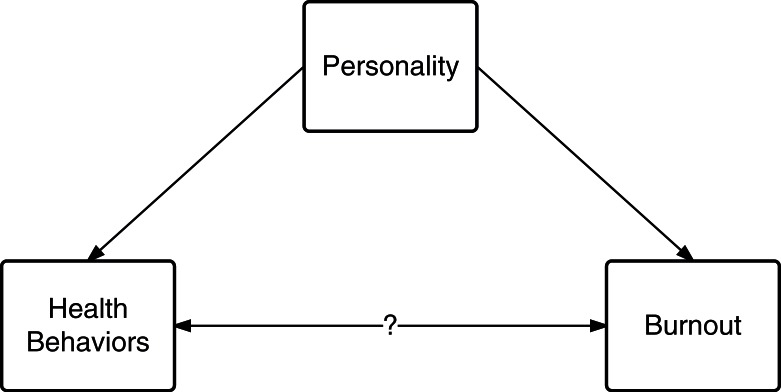
A basic schematic representation of the relationship between personality, burnout, and health behaviors. Notice that the three criteria of a confounding effect of personality on the relationship between health behaviors and burnout are applicable: 1) personality is a risk factor for burnout, independent of the putative risk factor (health behaviors), 2) personality is associated with putative risk factor (health behaviors), and 3) personality is not in the causal pathway between health behaviors and burnout.

**Fig. 2:**
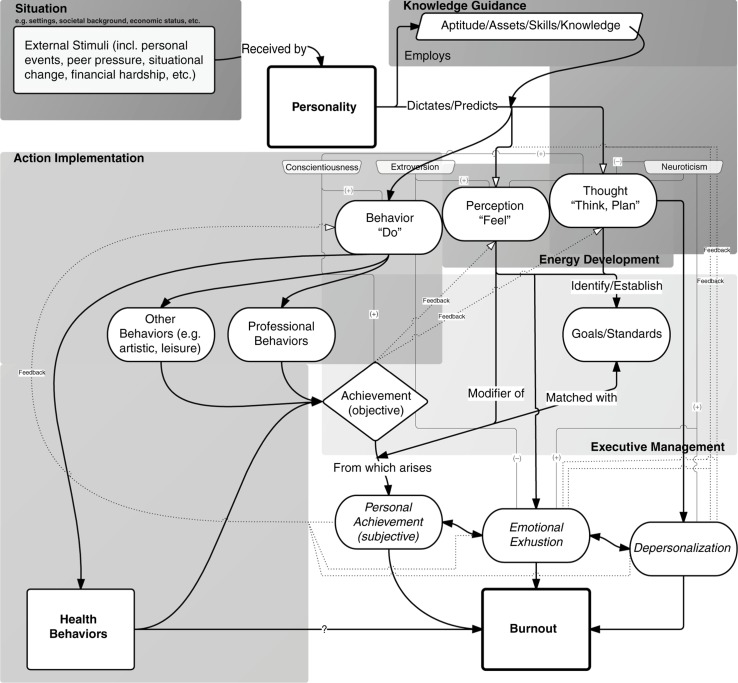
Personality-burnout model: A more detailed schematic representation of the interplay between personality, health behaviors, and burnout. Personality, by definition, would incorporate elements that are predictive of behaviors (including health-oriented behaviors). Should health behaviors independently be predictive of burnout, one can appreciate how personality, through a more comprehensive incorporation of predictive elements would represent a better prediction model of burnout. The background boxes (in shades of gray) indicate the functional areas of Mayer's Personality Systems Framework that correspond to each of the model’s components that are contained within these boxes. Three constructs of the Big Five Personality Trait Model (conscientiousness, extroversion, and neuroticism) are shown to exemplify how such background personality traits could positively (+) or negatively (−) influence the various parts of the pathway between external stimuli and burnout.

It seems that a third dimension to this issue exists (Fig. 1). Burnout, in a sense an adjustment disorder, is the product of the interaction between external stimuli and internal capabilities (or perceptions thereof) (6, 13–15); whether it be stress from academic challenges, self-set goals and expectations, learning climate, institutional culture, extracurricular demands, personal-life events, financial debt, discrimination, etc. (13, 15, 16); shaping the outcomes of such outer–inner interactions lies fundamentally in one's personality, which functions as the recipient and coordinator of the human inner mental systems that utilize opportunities and cope with difficulties encountered in life (Fig. 2) (17).

## Personality's effect in theory and context

Although no universally-accepted definition of personality exists, a particularly informative description of personality, which incorporates a common theme across the different views of personality in the literature, is that of Larsen and Buss:

“Personality is the set of psychological traits and mechanisms within the individual that are organized and relatively enduring and that influence his or her interactions with, and adaptations to, the intrapsychic, physical, and social environments.” (18)

In reality, applying a ‘psychobehavioral trait check’ to medical students can identify two opposing but mind-enlightening clusters of attributes: self-discipline, poise, dutifulness, proactivity, patience, and orderliness on one side and inattentiveness, impulsivity, negligence, passivism, and disorganization on the other. Possession of the first group of attributes allows one's knowledge, assets, and aptitude to be employed in the achievement of success in academia and maintenance thereof (Fig. 2) (19–21). Similar to the prerequisites of academic success, one's ability to initiate and sustain healthy behavior (e.g., daily exercise) requires comparable determination, discipline, and patience – all of which are largely dictated by personality traits (22, 23); the chances of a self-disciplined student, for example, to comply with the norms of desired health behaviors are arguably much greater than those of impulsive, unconcerned, and undisciplined counterparts.

Besides, the way perceptions of potential obstacles are composed, a key determinant of which is personality, can—in itself—empower a student with (or alternatively deprive the student of) the psychological grounds required to overcome encountered hindrances, regardless of their nature or the context in which they occur (24). Hardiness, a personality quality, for example, would be expected to protect against burnout. It delivers its protective effects by leading perceptions toward viewing stressors as challenges rather than threats, resulting in the resolution of a potential psychological disturbance (25). Therefore, taking a holistic view of the topic, one can understand the hypothetical basis of personality's influence on the accomplishment and sustainability of success in academic as well as lifestyle matters.

## The existing evidence

In addition to the conceivable theoretical foundation, the current body of evidence suggests the involvement of personality in both health behaviors (26, 27) and burnout (24, 28, 29). A 4.5-year longitudinal follow up of a representative sample of around 2000 Black and White US adolescents found certain personality factors to be related to risky health behaviors (such as alcohol consumption, tobacco use, and violence) and educational underachievement (30). Another 6-year, multicenter, admission-to-graduation longitudinal follow-up of medical students identified certain personality traits (e.g., neuroticism) as significant risk factors for experiencing highlevels of stress (31). Reports have also suggested the persistence of personality's effect on burnout beyond medical school (10, 29). A 12-year longitudinal study of UK medical graduates identified personality as a significant determinant of stress perception and eventual burnout (24). Likewise, McCranie et al. showed a clear correlation between the scores of maladaptive personality traits (i.e., “low self-esteem, feelings of inadequacy, dysphoria and obsessive worry, passivity, social anxiety, and withdrawal from others”) and high levels of burnout (29).

Therefore, personality, broadly defined, does exert influence on burnout. Of note, this influence seems to remain applicable when examined under various theoretical frameworks of personality. In their meta-analysis that included several chief personality constructs (e.g., core-self-evaluation, affectivity, proactivity, and the Five-Factor Model), Alarcon et al. showed the consistent relatedness of personality to the three components of burnout: EE, PA, and depersonalization (25). Similarly, using constructs from Cloninger's psychobiological model, primary evidence supports the existence of personality's influence on burnout (32) – a link which may well be embedded within basic coding blueprints of the human brain (33).

On the other hand, health behaviors seem to be less involved in burnout. A study evaluating the effect of an incentivizing exercise program at Mayo Clinic on physical activity and burnout found no significant difference in burnout levels between participants and non-participants despite the significant increase in meeting the U.S. Department of Health and Human Services’ recommendations for physical activity and exercise in the participants' cohort (34). This could be explained by the fact that physical activity, although may temporarily improve mental health and the perceived quality of life (5), does not address the underlying evoking factor (i.e., stressor) and accordingly plays a limited role in stress relief (35). In fact, on the contrary to what was believed about the positive effect of exercise on mental health, recent systematic reviews and meta-analyses have shown the effect size to be consistently small in rigorous study designs (36–38), with larger effects only seen in methodologically weaker reports (37). Given the involvement of personality in burnout and health behaviors, along with the limited impact of physical activity on burnout, personality may well be a confounder in the observed association between health behaviors and burnout (Fig. 1).

## Conclusion

With that in mind, recognizing personality's influence on health-oriented behaviors on the one hand, and its contribution to burnout on the other hand, may aid in identifying the scaffold around which our conceptual understanding of burnout can be constructed (Fig. 2). The existing evidence indicates personality's contribution to the likelihood of success in ordinary lifestyle and professional matters; those who are capable of facing challenges of sustaining healthy behaviors are likely to confront academic difficulties with the needed resoluteness and resilience, and thus, are somewhat less likely to experience burnout. This favors the hypothesis that health behaviors’ predictive capacity of burnout lies within its reflection of personality rather than a direct causality. Therefore, adjusting for potential confounding variables such as personality traits may be needed in the identification of the effect of health behaviors *per se* on the occurrence and development of burnout.
